# Emergence of high-level aztreonam–avibactam and cefiderocol resistance following treatment of an NDM-producing *Escherichia coli* bloodstream isolate exhibiting reduced susceptibility to both agents at baseline

**DOI:** 10.1093/jacamr/dlae141

**Published:** 2024-09-05

**Authors:** Ghady Haidar, Ellen G Kline, Georgios D Kitsios, Xiaohong Wang, Eun Jeong Kwak, Anthony Newbrough, Kelly Friday, Kailey Hughes Kramer, Ryan K Shields

**Affiliations:** Division of Infectious Diseases, University of Pittsburgh School of Medicine, Pittsburgh, PA, USA; Center for Innovative Antimicrobial Therapy, Division of Infectious Diseases, UPMC, Pittsburgh, PA, USA; Division of Infectious Diseases, University of Pittsburgh School of Medicine, Pittsburgh, PA, USA; Division of Pulmonary, Allergy, Critical Care and Sleep Medicine, University of Pittsburgh School of Medicine, Pittsburgh, PA, USA; Center for Medicine and the Microbiome, Division of Pulmonary, Allergy, Critical Care and Sleep Medicine, UPMC, Pittsburgh, PA, USA; Division of Pulmonary, Allergy, Critical Care and Sleep Medicine, University of Pittsburgh School of Medicine, Pittsburgh, PA, USA; Center for Medicine and the Microbiome, Division of Pulmonary, Allergy, Critical Care and Sleep Medicine, UPMC, Pittsburgh, PA, USA; Division of Infectious Diseases, University of Pittsburgh School of Medicine, Pittsburgh, PA, USA; Division of Infectious Diseases, University of Pittsburgh School of Medicine, Pittsburgh, PA, USA; Division of Infectious Diseases, University of Pittsburgh School of Medicine, Pittsburgh, PA, USA; Division of Infectious Diseases, University of Pittsburgh School of Medicine, Pittsburgh, PA, USA; Division of Infectious Diseases, University of Pittsburgh School of Medicine, Pittsburgh, PA, USA; Center for Innovative Antimicrobial Therapy, Division of Infectious Diseases, UPMC, Pittsburgh, PA, USA; Antibiotic Management Program, Division of Infectious Diseases, UPMC, Pittsburgh, PA, USA

## Abstract

**Background:**

Cefiderocol (FDC) or ceftazidime-avibactam with aztreonam (CZA-ATM) are frontline agents for New Delhi metallo-β-lactamase (NDM)-producing Enterobacterales; however, clinical data are scarce, and mechanisms of treatment-emergent resistance are ill-defined. Our objectives were to characterize serial isolates and stool microbiota from a liver transplant recipient with NDM-producing *Escherichia coli* bacteraemia.

**Methods:**

Isolates collected pre- and post-CZA–ATM treatment underwent broth microdilution susceptibility testing and whole-genome sequencing. Longitudinal stool collected during CZA–ATM therapy underwent metagenomic sequencing (Nanopore MinION).

**Results:**

The baseline isolate exhibited elevated MICs for ATM–AVI (16/4 µg/mL) and FDC (8 µg/mL). Posttreatment, a rectal surveillance isolate exhibited high-level resistance to ATM–AVI (> 128/4 µg/mL) and FDC (32 µg/mL). Both isolates belonged to ST361 and harboured WT *bla*_NDM-5_. The baseline isolate contained wild type (WT) *bla*_CMY-145_ and mutations in *ftsI* (which encodes PBP3), including a YRIN insertion at residue 338 and the non-synonymous substitutions Q227H, E353K and I536L. The posttreatment isolate harboured new mutations in *ftsI* (A417 V) and *bla*_CMY-145_ (L139R and N366Y). Analysis of four stool samples collected during CZA–ATM treatment revealed high *E. coli* abundance. *E. coli* relative abundance increased from 34.5% (first sample) to 61.9% (last sample).

**Conclusions:**

Baseline mutations in *ftsI* were associated with reduced susceptibility to ATM–AVI and FDC in an ST361 NDM-5-producing *E. coli* bloodstream isolate. High-level resistance was selected after CZA–ATM treatment, resulting in new *ftsl* and *bla*_CMY-145_ mutations. These findings underscore the need for ATM–AVI susceptibility testing for NDM producers, and the potential for PBP3 mutations to confer cross-resistance to ATM–AVI and FDC, which can emerge after CZA–ATM treatment.

## Introduction

The novel β-lactam/β-lactamase inhibitors ceftazidime-avibactam (CZA), meropenem–vaborbactam and impenem–relebactam have revolutionized the management of carbapenem-resistant Enterobacterales (CRE) that produce *Klebsiella pneumoniae* carbapenemases (KPCs).^1^ Unfortunately, these agents lack activity against Enterobacterales carrying metallo-β-lactamases (MBLs), including New Delhi metallo-β-lactamases (NDMs), limiting therapeutic options for these organisms. In recent years, two frontline options have emerged for MBL-producing CRE. The first is combination therapy with CZA and aztreonam (CZA–ATM), which is active against MBL-producing CRE because ATM is not hydrolyzed by MBLs, and avibactam (AVI) protects aztreonam from hydrolysis by serine β-lactamases often present in MBL-producing CRE.^1^ ATM–AVI is not yet available (NCT03329092), necessitating the use of combination CZA–ATM.^1^ Cefiderocol (FDC), a novel siderophore antibiotic, demonstrates excellent *in vitro* activity against these pathogens.^1^ Accordingly, either CZA–ATM or FDC is recommended for serious infections due to MBL-producing CRE.^1^ However, clinical experience remains limited, and surveillance studies have demonstrated that not all NDM-producing CRE are susceptible to ATM–AVI or FDC.^2–9^ Moreover, treatment-emergent resistance to either agent has not been widely reported.^10,11^

Herein, we report a liver transplant recipient who developed bacteraemia on post-operative day (POD)-6 with an *Escherichia coli* isolate that produced NDM-5 and CMY-145, with reduced susceptibility to ATM–AVI and FDC. Bacteraemia resolved with CZA–ATM treatment, but the patient later developed rectal colonization with *E. coli* exhibiting high-level resistance to both agents. We sought to determine the mechanisms of baseline and treatment-emergent resistance and to characterize the stool microbiota during and after CZA–ATM treatment.

## Case presentation

A 48-year-old male with alcoholic cirrhosis underwent a living-donor liver transplant from his daughter under basiliximab induction. He received tacrolimus, mycophenolate and prednisone; standard post-operative prophylaxis consisted of trimethoprim–sulfamethoxazole, acyclovir, fluconazole and 5 days of ampicillin-sulbactam. The patient was originally from India and had immigrated to the USA 25 years prior. He visited India periodically; his last trip was 1 year pre-transplant. His daughter had last travelled to India 4 years pre-donation.

**Figure 1.**
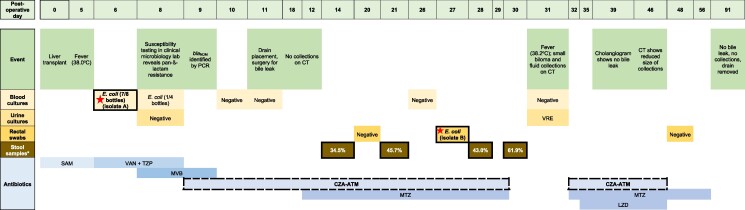
Sequence of events. *Stool samples obtained for research only (metagenomic sequencing); corresponding percentages represent relative *E. coli* abundance in stool. Boxes with thick solid borders denote days of specimen collection; red stars denote the *E. coli* isolates that underwent additional susceptibility testing and whole-genome sequencing (isolates-A and B). Boxes with thick dashed borders represent days of CZA–ATM administration. CT, computed tomography; CZA–ATM, ceftazidime–avibactam plus aztreonam; LZD, linezolid; MTZ, metronidazole; MVB, meropenem–vaborbactam; SAM, ampicillin–sulbactam; TZP, piperacillin–tazobactam; VAN, vancomycin; VRE, vancomycin resistant *Enterococcus*.

The patient’s intra-operative course was uncomplicated. On post-operative day (POD)-5, he developed a fever (38.0°C), but was otherwise stable. Blood cultures were collected on POD-6 (Figure 1), and he was started on vancomycin and piperacillin-tazobactam. Cultures grew *E. coli* in seven of eight bottles. Susceptibility testing (POD-8) revealed resistance to all routinely tested agents, except aminoglycosides. Carbapenemase production was identified using the modified carbapenem activation method.^12^ Reflex susceptibility testing for CZA and meropenem–vaborbactam was requested, and the patient was switched to meropenem–vaborbactam (4 g IV every 8 h) for presumed KPC–*E. coli*. Blood cultures on POD-8 remained positive. Susceptibility testing results revealed that the initial *E. coli* was resistant to both CZA and meropenem–vaborbactam. An in-house multiplex PCR assay detected *bla*_NDM_ on POD-9; these results were shared with the clinical team, prompting a switch to CZA 2.5 g IV every 8 h and aztreonam 2 g IV every 8 h.

Six sets of follow-up blood cultures were negative. A bile leak was managed surgically. Computed tomography (CT) did not show intra-abdominal collections. The patient received CZA–ATM for 20 days, through POD-29. On POD-31, he became febrile to 38.2°C but remained stable. Blood and urine cultures were negative for *E. coli*, although the urine culture grew vancomycin-resistant *Enterococcus* (Figure 1). A CT scan showed a biloma and fluid collections, which were not amenable to procedures. He received an empiric 2-week course of CZA–ATM, through POD-46; collections decreased in size. On POD-91, imaging revealed no biliary leak and resolution of the collections. The patient improved and was discharged, with no relapse of this infection nearly 3 years post-transplant.

Cultures from CRE rectal surveillance swab, which are performed weekly for routine surveillance on all transplant recipients (chromogenic agar, Hardy Diagnostics, Santa Maria, CA, USA), were negative on POD-20, positive for *E. coli* on POD-29 and negative on POD-48 (Figure 1). A baseline pre-transplant surveillance swab was not available.

## Methods

To characterize *E. coli* isolates and stool microbiota, the patient was enrolled in a prospective observational study at UPMC (STUDY20090146). *E. coli* causing bacteraemia (isolate-A) and rectal colonization (isolate-B) were collected (Figure 1). Stool samples were not available prior to bacteraemia from the patient or the patient’s daughter. Susceptibility testing was performed by broth microdilution in triplicate according to the CLSI guidelines.^13^ ATM–AVI was tested as a surrogate for CZA plus ATM, and the ATM breakpoint was used to define susceptibility.^14^ We performed whole genome sequencing (WGS) as previously described.^15^ Stool from POD-14, 21, 28 and 30 underwent metagenomic sequencing using the Oxford Nanopore Technologies MinION Mk1c device as previously described^16^ (Supplementary Methods, available as Supplementary data at *JAC-AMR* Online).

## Results

Both isolates exhibited high-level resistance against aztreonam, ceftazidime and CZA (Table 1). Isolate-A (pre-CZA–ATM) was non-susceptible to ATM–AVI (MIC 16 µg/mL) and intermediately susceptible to FDC (MIC 8 µg/mL). Adding AVI did not lower the FDC MIC. Isolate-B, collected 21 days after isolate-A, exhibited high-level resistance to ATM–AVI (MIC > 128/4 µg/mL). FDC and FDC–AVI MICs increased by 4- and 2-fold, respectively (Table 1).

**Table 1.** Results of whole-genome sequencing and susceptibility testing of *E. coli* isolates

Isolate	Source	Days since transplant	Days since start of CZA–ATM	Whole-genome sequencing results									MICs (BMD, µg/mL)					
				ST	Plasmid types	NDM-5	CirA	CMY-145	PBP3	*marR*	*aroP*	*envZ*	FDC	FDC–AVI	ATM	ATM–AVI	CAZ	CZA
A	Blood	+ 6	− 3	361	IncFIA, IncFII	WT	WT	WT	338insYRIN, Q227H, E353K, I536L	S103G, H137Y	A171G, S184T	V25A, M446T	8	8/4	> 32	16/4	> 128	> 128/4
B	Rectal swab	+ 27	+ 18	361	IncFIA, IncFII	WT	WT	L139R^a^, N366Y^a^	338insYRIN, Q227H, E353K, I536L, A417V^a^	S103G, H137Y	A171G, S184T	V25A, M446T	32	16/4	> 32	> 128/4	> 128	> 128/4
																		

The isolates exhibited 19 core SNP differences. Isolate-A was the baseline isolate causing bacteraemia, with no prior exposure to CZA–ATM or FDC. Isolate-B was collected from a surveillance rectal swab, following treatment with CZA–ATM.

ATM, aztreonam; ATM–AVI, aztreonam, avibactam; BMD, broth microdilution; CAZ, ceftazidime; CZA, ceftazidime–avibactam; CZA–ATM, ceftazidime–avibactam plus aztreonam; FDC, cefiderocol; FDC–AVI, cefiderocol, avibactam; ins, insertion; MIC, minimum inhibitory concentration; NA, not applicable, collected 3 days before CZA–ATM started; PBP3, penicillin-binding protein 3, ST, sequence type; WT, wild type.

^a^New mutations that emerged following CZA–ATM therapy.

Both isolates belonged to sequence type (ST) 361^3^ and varied by 19 core SNP differences (Table 1). Isolate-A carried wild type (WT) *bla*_NDM-5_, *bla*_CMY-145_ and *cirA*. PBP3 (encoded by *ftsl)* contained a YRIN insertion at residue 338 and non-synonymous substitutions Q227H, E353K and I536L (Table 1). Isolate-B also carried WT *bla*_NDM-5_ and *cirA*, but exhibited new mutations in PBP3 (A417V) and CMY-145 (L139R and N366Y). No other new mutations were identified in genes previously implicated in FDC resistance, including *envZ*, *marR* and *aroP*^11^ (Table 1).

Stool metagenomic sequencing from POD-14, 21, 28 and 30 (during CZA–ATM therapy) revealed a high relative abundance of *E. coli* in all samples (34.5%, 45.7%, 43.0% and 61.9%, respectively) (Figure 1 and supplementary Figure A). There was significantly increased relative abundance of *E. coli* from POD-14 to POD-30 (χ^2^  *P* = 0.0004). *bla*_NDM-5_ was present in all four samples (205, 333, 195 and 437 read alignments, respectively) (supplementary Figure B). However, when normalized by the most abundant resistance gene in all samples (*bla*_CMY-59_), there was no evidence of increased normalized abundance for *bla*_NDM-_5 gene (supplementary Figure C). Whole metagenomic sequencing directly from stool did not identify *fstl* or *bla*_CMY-145_ (Supplementary Results).

## Discussion

We report a living-donor liver transplant recipient from India who developed early post-transplant bacteraemia caused by NDM-5-producing ST361 *E. coli* that was non-susceptible to ATM–AVI and FDC despite no prior exposure to either agent. Baseline non-susceptibility was presumably mediated by mutations in PBP3 and co-production of the AmpC β-lactamase CMY-145.^2–9,17^ Bacteraemia resolved following CZA–ATM, but the patient became colonized with *E. coli* exhibiting higher-level resistance, with new mutations in PBP3 and CMY-145. Our findings highlight a concerning NDM-producing CRE clone exhibiting resistance to the frontline anti-MBL agents, CZA–ATM and FDC.^1^ These data corroborate surveillance studies showing that resistance to these agents may be an emerging threat.^2–9^

Treatment with CZA–ATM results in improved survival compared with colistin-based therapies for patients with MBL-producing CRE bacteraemia.^18^ Baseline and treatment-emergent resistance has not been widely reported in the limited real-world experience.^10,11^ Indeed, NDM-producing CRE with resistance to ATM–AVI are rare, but certain clones exhibit reduced susceptibility.^2,4–9^ Among the most commonly reported high-risk ATM–AVI non-susceptible clones are those that carry mutations in PBP3,^2,4,5,7–9^ specifically a four-amino-acid insertion (YRIN/K) at residue 333 of PBP3.^2,4,5,7–9^ This mutation significantly reduces aztreonam’s affinity towards PBP3,^2,5^ and we identified the same insertion at residue 338 in isolate-A recovered from our patient (Table 1). The presence of the YRIN/K insertion in PBP3 alone, however, may not be sufficient to confer high-level ATM–AVI resistance.^2,4–9^ Other PBP3 substitutions identified in isolate-A (Q227H, E353K and I536L) have also been implicated in reduced susceptibility to ATM–AVI, typically with YRIN/K insertions.^7,9^ Higher-level resistance may also coincide with the presence of WT or variant cephalosporinases like CMY-42 or CMY-145,^2,4–6,9^ the latter of which was found in both isolate-A and isolate-B (Table 1).

Isolate-B developed a > 8-fold increased ATM–AVI MIC compared with isolate-A. We hypothesize that this was mediated by new mutations in PBP3 and CMY-145 that were selected during CZA–ATM therapy. Isolate-B also carried a new A417V substitution in PBP3, which lies opposite the PBP3 active site and hinders substrate binding, since valine is bulkier than alanine.^3,8^ New L139R and N366Y substitutions in CMY-145 may have further contributed to ATM–AVI resistance due to increased ATM hydrolysis or weaker inhibition by AVI.^4,6^

FDC is another key option for MBL-producing CRE, resulting in numerically lower all-cause mortality versus other therapies in clinical trials of patients whose isolates exhibited FDC MICs ≤4 µg/mL.^19^ By contrast, our patient’s baseline isolate demonstrated a FDC MIC of 8 µg/mL. As with ATM–AVI, the mechanisms of FDC resistance are multifactorial and likely related to mutations in PBP3 and/or CMY.^3,17,20^ For example, the 333-YRIN insertion in PBP3 and the substitutions Q227H, E349K, I532L and A412V [identified in isolate-A (338 insertion) and/or isolate-B, Table 1] have been associated with FDC resistance.^3,20^ Co-existence of CMY-145^20^ (identified in both our isolates) and mutations within and increased expression of CMY-type AmpC β-lactamases^17^ have also been documented in MBL-producing isolates with FDC resistance. The extent to which the new L139R and N366Y mutations in CMY-145 contributed to increased FDC resistance in isolate-B is unknown. Increased expression of *bla*_NDM-5_ has been previously associated with FDC resistance in a single case, but unlike our case, PBP3 mutations and *bla*_CMY_ were not detected.^11^ Mutations in other genes, including *envZ*, *marR* and *aroP*, have also been reported following FDC treatment of NDM-producing *E. coli*, but did not vary from baseline in our case. Finally, although mutations in the iron transporter gene *cirA* have been associated with FDC resistance,^3,20^ these were not identified in either case.

Since our patient’s bacteraemia resolved with CZA–ATM treatment, and because rectal CRE colonization predicts infection post-transplant,^21^ we hypothesized that interrogating the stool microbiome would reveal a reduction in the abundance of *E. coli*. Instead, analysis of serial stool samples showed a high abundance of *E. coli* and persistent detection of *bla*_NDM-5_ from POD-18 to POD-30 (Supplementary Figures/Results). Interestingly, the abundance of *E. coli* during CZA–ATM therapy increased, which may be due to selection pressure to the commensal stool harbouring an *E. coli* population that had developed high-level resistance to ATM–AVI. Further studies are needed to longitudinally characterize the stool microbiota in CRE infection after transplantation and determine the variables associated with acquisition and resolution of CRE colonization. These studies are currently underway at our centre.

Our report has some limitations, including the fact that our findings are based on a single patient. Nonetheless, our experience corroborates other reports. In addition to the previously mentioned case,^11^ Simner *et al.*^10^ also described another patient who underwent kidney transplant in India and then developed pyelonephritis due to an ST167, NDM-5-producing *E. coli* with reduced susceptibility to ATM–AVI and FDC. Resistance was attributed to the presence of CMY-59 and a YRIN insertion at PBP3 position 338. In our case, the specific contribution of new PBP3 and CMY mutations resulting in higher-level resistance requires future validation. Although we characterized the stool microbiome over a 2-week period, the absence of stool pre-transplant and at additional timepoints post-transplant prevents us from making other conclusions. In addition, sparse sampling from the patient by peri-rectal surveillance swabs limits our ability to comment on the duration of colonization or possibility of decolonization following treatment. Finally, we could not obtain biospecimens from the patient’s daughter to determine whether the *E. coli* was donor-derived.

Our experience and recent reports indicate that the activity of ATM–AVI and FDC should not be presumed against NDM-producing *E. coli* and that therapy should be guided by susceptibility testing whenever possible. The greatest vulnerability to both these frontline options appears to be the presence of specific PBP3 mutations and CMY β-lactamases; however, other mechanisms of resistance have been reported.^11^ Future studies should also evaluate whether optimized CZA–ATM dosing may overcome reduced baseline susceptibility or suppress the emergence of resistance,^22^ the clinical effectiveness of ATM–AVI against diverse MBL-CRE, and the role of novel agents^10^ such as FEP–zidebactam, FEP–taniborbactam and FDC–xeruborbactam. Complimentary or enhancer effects of PBP2 inhibition with zidebactam or durlobactam also merit further investigation.^23^ Finally, we propose that the role of microbiota-directed therapies like faecal microbiota transplantation could be explored for patients known to be colonized with extensively drug-resistant pathogens.

## Supplementary Material

dlae141_Supplementary_Data

## Transparency declarations

G.H. is a recipient of research grants from Allovir, Karius, Regeneron, the Cystic Fibrosis Foundation and AstraZeneca, also serves on the scientific advisory boards of Karius, AstraZeneca, Pfizer, REDQueen and SNIPR BIOME and has received honoraria from MDOutlook, PeerView Institute for Medical Education and PRIME Inc. G.D.K. has received research funding from Karius, Inc. and Pfizer, Inc., both unrelated to this work. R.K.S. has served as a consultant for Allergan, Cidara, Shionogi, Menarini, Melinta, Merck, Entasis, Utility and Venatorx and has received investigator-initiated funding from Merck, Melinta, Shionogi and Venatorx. G.H., G.D.K. and R.K.S. have no other items to declare. All other authors report no potential conflicts of interest.

## Supplementary data

Supplementary Figure A–C is available as Supplementary data at *JAC-AMR* Online.
