# Supplementary material for: Emergence of high-level aztreonam–avibactam and cefiderocol resistance following treatment of an NDM-producing Escherichia coli bloodstream isolate exhibiting reduced susceptibility to both agents at baseline
Source: JAC Antimicrob Resist. 2024 Sep 5;6(5):dlae141. doi: 10.1093/jacamr/dlae141 (PMC11375572; doi:10.1093/jacamr/dlae141)
Supplement: dlae141_Supplementary_Data [file dlae141_supplementary_data.docx]

**Supplementary materials:**

**1. Supplementary methods**

**2. Supplementary figures**

**3. Supplementary results**

**1. Supplementary methods:**

***Metagenomic sequencing of stool samples:***

We extracted genomic DNA from stool samples with the DNeasy Powersoil kit from Qiagen. We performed metagenomic Nanopore sequencing of extracted DNA with a rapid PCR barcoding kit (SQK-RPB004) on the MinION device (Oxford Nanopore Technologies-ONT, Oxford, UK) for 24h. We analyzed microbial metagenomic sequences with the EPI2ME platform (ONT) and the “What’s In My Pot” [WIMP] workflow to quantify abundance of microbial species. WIMP is an EPI2ME workflow for taxonomic classification of nanopore sequencing reads, classifying each sequence present in FASTQ files. We filtered FASTQ files with a mean quality (q-score) below a minimum threshold of 7. For reads above the quality threshold, WIMP uses the Centrifuge classification engine to assign each read to a taxon in the NCBI taxonomy. The Centrifuge classification results are then filtered and aggregated to calculate and report counts of reads at the species rank. For reads without a reliable assignment at the species rank, higher ranks of the taxonomy tree are used for the assignment. If no placement is reliable enough (below a scoring threshold), the sequence is labeled as "Unclassified".

**N of high-quality reads aligned to *bla*_NDM-5_ by Nanopore metagenomics in each stool sample normalized to N of *bla*_CMY-59_ in each sample**

**Total N of high-quality reads aligned to *bla*_NDM-5_ by Nanopore metagenomics in each stool sample**

*bla*_CMY-59_

*bla*_NDM-5_

**A.**

**2. Supplementary Figures (legend on next page)**

**B.**

**C. Proportion of *bla_NDM-5_* reads over *bla*_CMY-59_ *in stool samples***

**Post-operative day**

**Post-operative day**

**Post-operative day**

**Total N of high-quality reads by Nanopore metagenomics of each stool sample**

***E. coli abundance in stool versus other DNA organisms***

**45.7%**

**43.0%**

**61.9%**

**34.5%**

***Supplementary Figure legend:***

**61.9%**

**43.0%**

**45.7%**

**34.5%**

Nanopore metagenomics results of four stool samples available in the post-operative period.

**A.** *E. coli* abundance in each sample in relationship to other DNA organisms (bacteria, fungi, and viruses) in each sample by metagenomic sequencing. Height of each bar plot corresponds to the total number of high-quality reads produced by each sequencing run that were successfully classified to DNA organisms. The red shaded part of each plot corresponds to the total N of reads classified to *E. coli*, with annotated relative abundance proportion (%) in white fonts. There was a statistically significant increase in *E. coli* relative abundance from the first (Day 14) to the last (Day 30) sample.

**B**. Total N of reads in each sample that were mapped to the *bla*_NDM-5_ resistance gene according to the Comprehensive Antibiotic Resistance Database (CARD database) via the Antimicrobial Resistance Pipeline of Epi2Me.

**C.** Normalized N of reads in each sample mapped to the *bla*_NDM-5_ (red) by the total N of reads mapped to the most abundant resistant gene in all samples, which was *bla*_CMY-59_ grey.

**3. Supplementary results:**

Stool metagenomic sequencing from POD-14, 21, 28, and 30 (during CZA-ATM therapy) revealed high relative abundance of *E. coli* in all samples (34.5%, 45.7%, 43.0%, and 61.9%, respectively) (**Supplementary Figure A**). There was significantly increased relative abundance of *E. coli* from POD-14 to POD-30 (chi-square p=0.0004). *bla*_NDM-5_ was present in all four samples (205, 333, 195 and 437 read alignments, respectively) (**Supplementary Figure B**). However, when normalized by the most abundant resistance gene in all samples (*bla*_CMY-59_), there was no evidence of increased normalized abundance for *bla*_NDM-5_ gene (**Supplementary Figure C)**.

Stool metagenomic sequencing did not detect *bla*_CMY-145_ or any *fstl* genes (encoding PBP-3) which our patient’s isolates harbored. By contrast, metagenomic sequencing directly from stool detected several genes in the *bla*_CMY_ family, with *bla*_CMY-59_ and *bla*_CMY-42_ being the most abundant. However, WGS indicated that they were not present in our patient’s *E. coli* isolate. Potential explanations for the discrepancies between *E. coli* WGS and stool metagenomics sequencing results include a) the possibility that the *bla*_CMY-59_ and *bla*_CMY-42_ genes were present not in the NDM-5-*E. coli* but rather in other stool organisms in the sequenced metagenome (e.g., other Enterobacterales harboring *bla*_CMY_ genes), which prevented us from definitively assigning these gene to the NDM-5-*E.coli,* b) the possibility that *blaCMY*-_145_ and *fstl* were present in low abundance in the NDM-5-*E. coli* and thus could not be detected by whole metagenome sequencing directly from stool, c) issues arising from stool specimen processing, and/or d) issues regarding the Comprehensive Antibiotic Resistance Database (CARD database). We could not assess for mutations in *bla*_CMY-59_ and *bla*_CMY-42_, as the coverage and estimated accuracy per the Epi2ME software were limited due to our approach of performing rapid metagenomic sequencing of DNA extracted directly from samples and not clinical isolates. For example, the accuracy for *bla*_CMY_-59 ranged from 89.9% to 91.8%.
